# Closed-Loop Vagus Nerve Stimulation for the Treatment of Cardiovascular Diseases: State of the Art and Future Directions

**DOI:** 10.3389/fcvm.2022.866957

**Published:** 2022-04-07

**Authors:** Matteo Maria Ottaviani, Fabio Vallone, Silvestro Micera, Fabio A. Recchia

**Affiliations:** ^1^Institute of Life Sciences, Scuola Superiore Sant’Anna, Pisa, Italy; ^2^Department of Excellence in Robotics and Artificial Intelligence, The BioRobotics Institute, Scuola Superiore Sant’Anna, Pisa, Italy; ^3^Bertarelli Foundation Chair in Translational Neural Engineering, Center for Neuroprosthetics, Institute of Bioengineering, Ecole Polytechnique Federale de Lausanne, Lausanne, Switzerland; ^4^Fondazione Toscana Gabriele Monasterio, Pisa, Italy; ^5^Department of Physiology, Cardiovascular Research Center, Lewis Katz School of Medicine at Temple University, Philadelphia, PA, United States

**Keywords:** autonomic nervous system, vagus nerve stimulation, cardiovascular diseases, neural decoding, closed-loop

## Abstract

The autonomic nervous system exerts a fine beat-to-beat regulation of cardiovascular functions and is consequently involved in the onset and progression of many cardiovascular diseases (CVDs). Selective neuromodulation of the brain-heart axis with advanced neurotechnologies is an emerging approach to corroborate CVDs treatment when classical pharmacological agents show limited effectiveness. The vagus nerve is a major component of the cardiac neuroaxis, and vagus nerve stimulation (VNS) is a promising application to restore autonomic function under various pathological conditions. VNS has led to encouraging results in animal models of CVDs, but its translation to clinical practice has not been equally successful, calling for more investigation to optimize this technique. Herein we reviewed the state of the art of VNS for CVDs and discuss avenues for therapeutic optimization. Firstly, we provided a succinct description of cardiac vagal innervation anatomy and physiology and principles of VNS. Then, we examined the main clinical applications of VNS in CVDs and the related open challenges. Finally, we presented preclinical studies that aim at overcoming VNS limitations through optimization of anatomical targets, development of novel neural interface technologies, and design of efficient VNS closed-loop protocols.

## Introduction

Cardiovascular diseases (CVDs) still represent a major disease burden worldwide, despite advances in pharmacological treatments (1). Therefore, new therapeutical strategies are currently being investigated as an alternative to classical schemes. Among those, the solutions offered by Bioelectronic Medicine (BM) (2–5)—a new, highly interdisciplinary field incorporating neuroscience, engineering, and molecular medicine (4, 6)—are emerging as appealing candidates. The development of BM was inspired by the growing comprehension of the autonomic nervous system (ANS), which plays a key role in the control of whole-body homeostasis. Dysfunctions of the ANS are consequently implicated in the development and progression of many diseases, including those affecting the cardiovascular system (4, 7–13). BM utilizes this body of knowledge as a reference for the design of implantable devices (5) that modulate signals of the peripheral nervous system to visceral organs for therapeutic purposes (14–16).

The neural control of cardiovascular functions involves multiple interactions among central and peripheral components of the so-called “cardiac neuraxis” (17), which comprises the intrinsic cardiac nervous system, vagus nerves (VNs), intrathoracic sympathetic ganglia, spinal cord, brain stem, and multiple central regions up to the insular cortex. Acting together, these hierarchically organized functional units coordinate and regulate cardiac activity to preserve an adequate match between cardiac output and blood flow demand (18). Autonomic dysregulation at various levels of the cardiac neuroaxis, from central nuclei to peripheral effectors, is now recognized as a fundamental contributor to the progression of CVDs (1, 19). For instance, altered neural signals, such as the pathological activation of cardiac afferent neurons by acute myocardial ischemia and reperfusion, induce maladaptive responses such as sympathetic overdrive and parasympathetic withdrawal (autonomic imbalance) that in turn contribute to the development of systemic cardiovascular alterations (1, 19, 20). Therefore, the selective modulation of the cardiac neuraxis to achieve targeted control of cardiovascular functions has been proposed as a potentially impactful application of BM (21, 22). Peripheral nerves and ganglia of the ANS are attractive targets for BM for their favorable location for surgical interventions compared with deep and anatomically less characterized ANS centers of the CNS like the periaqueductal gray matter of the midbrain or hypothalamic nuclei (11, 23). In this perspective, the most widely studied intervention is represented by the electrical stimulation of the VN (VNS) (24, 25).

The VN (or X cranial nerve) is a paired asymmetric and the most extensively distributed nerve in the body, as well as a major component of the cardiac neuraxis (26). Sensory signaling through the VN plays a critical role in maintaining homeostasis of feeding, digestion, respiration, and cardiovascular functions (27). For this reason, VN neuromodulation is being tested as a potential therapeutical strategy for many pathological conditions, including CVDs (28). However, despite promising results achieved in preclinical studies (29), VNS is accompanied by side effects and still needs to be optimized for better exploitation of its full potential in the clinical setting. Herein we will review the state of the art of VNS for CVDs and discuss the current perspectives of VNS optimization. First, we will summarize the anatomy and physiology of the cardiac vagal system; then, we will describe the principles of VNS for CVDs and its main clinical applications, with related open challenges. Finally, we will review preclinical studies aimed at overcoming VNS limitations through optimization of anatomical targets, development of novel neural interface technologies, and design of efficient VNS closed-loop protocols. In the latter case, we will particularly focus on neural decoding strategies that aim at the identification of timely- and spatially selective feedback signals to drive VNS for CVDs properly (8, 30–32).

## Anatomy and Physiology of Vagus Nerve in the Cardiovascular System

### Gross Anatomy

**FIGURE 1:**
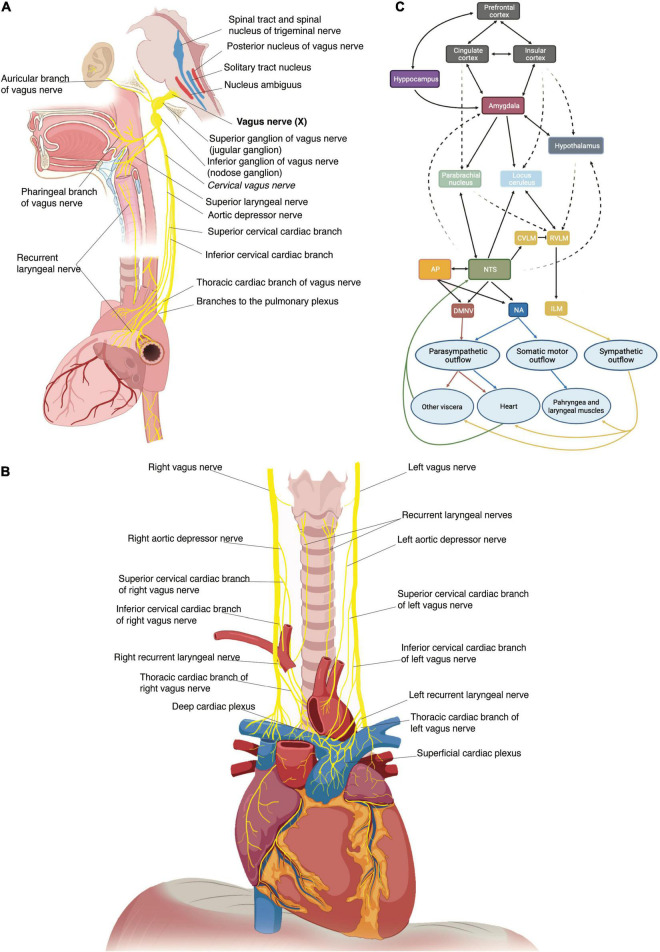
**(A)** Schematic representation of the origin of the vagus nerve from the medulla, its ganglia and its major branches at the cervical and thoracic levels. **(B)** Thoracic vagus nerves with cervical and thoracic cardiac branches to the deep and superficial cardiac plexi and recurrent laryngeal nerves. **(C)** Schematic representation of the central autonomic network with internuclei connections. An autonomic vagovagal loop comprises visceral inputs to the nucleus tractus solitarii (NTS) which then sends outputs to the dorsal motor nucleus (DMNV), rostral ventrolateral medullary (RVLM), and intermediate lateral medulla (ILM) to adapt autonomic balance to physiological demands. The cross-talk between the NTS and brain regions (hypothalamus, amygdala, cingulate cortex, insula, prefrontal cortex) engaged in neuroendocrine, affective, and cognitive regulation of behavior modulates this autonomic forebrain loop. AP, area postrema; NA, nucleus accumbens.

The VN originates bilaterally in the medulla as multiple filaments that extend toward the jugular foramen and then converge to form a single trunk. Within or just caudal to the jugular foramen are located the superior (jugular) and inferior (nodose) ganglia of the VN (Figure 1A) (26). At its emergence from the nodose ganglion, the VN can be anatomically divided into three segments along the rostro-caudal direction: cervical, thoracic and abdominal (26). The cervical VN generates multiple branches, including the superior cardiac and aortic branches (Figure 1A) (26, 33–36). Leaving the carotid sheath at the neck base, the VN enters the thorax, and it is referred to as the thoracic VN. Vagal cardiac branches include the superior and inferior cervical cardiac branches and the inferior or thoracic cardiac branch that originates from the thoracic VN (Figures 1A,B) (26, 37). The aortic branch or depressor nerve from the left VN contains afferent fibers innervating the aortic arch, while the one from the right VN innervates the bifurcation of the right brachio-cephalic trunk (35, 38). Within the mediastinum, the thoracic VN provides thoracic cardiac branches that are mainly observed between the aortic arch and the pulmonary arterial trunk and innervate the heart along the coronary arteries (37). Together with cardiac nerves from the sympathetic trunk, they contribute to form the cardiac plexus, which is usually divided into a superficial and a deep portion (Figure 1B). From a functional perspective, the right-sided nerves innervate mostly the sinoatrial node, while the left-sided nerves innervate mostly the atrioventricular node (36).

### Microscopic Anatomy

**FIGURE 2:**
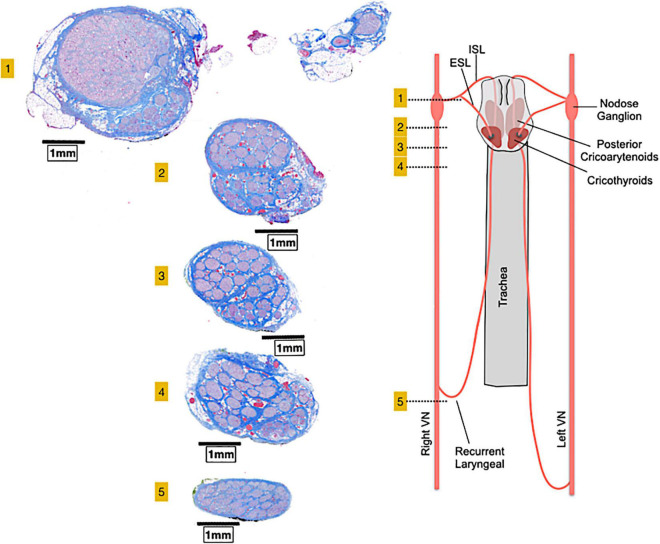
A bimodal arrangement, or vagotopy, can be observed in histological sections collected at various levels along the pig vagus nerve. Section 1 comprises the nodose ganglion and the superior laryngeal branch and show pseudo-unipolar cells grouped in a huge “fascicle” from which arises a smaller group of fascicles in sections 2, 3, and 4. Caudally to the recurrent laryngeal nerve (section 5), the bimodal architecture is no longer visible. ESL, external superior laryngeal nerve; ISL, internal superior laryngeal nerve, VN, vagus nerve. Adapted from Settell et al. (44).

Similar to other peripheral nerves, vagal fibers are grouped into a variable number of fascicles (39) with high variability among different species and even within the same species (40, 41). Several studies in human cadavers found the mean fascicles number of the cervical VN to oscillate between 5 and 10 (33, 40, 42, 43). The cervical VN in mice, rats, canines and non-human primates displays a less complex fascicular organization than in humans, typically consisting of 1–2 fascicles (43, 44). The porcine VN displays more fascicles than the human, containing 46 ± 10 and 43 ± 8 bundles at cervical and abdominal level, respectively (43, 44). In the somatic nervous system, all fascicles seem to conform a somatotopic organization (45, 46) and whether this occurs also in the ANS needs still to be assessed even if it is highly possible in complex nerves such as the VN (47). In fact, Settell et al. described a distinct bimodal organization of fascicles in the pig cervical VN. Specifically, they observed pseudounipolar cells aggregated in a large “fascicle” in nodose ganglion cross-sections and found a distinct group of fascicles arising from that large “fascicle” in caudal cross-sections of the cervical VN (Figure 2). This distinct organization of fascicles disappeared beyond the recurrent laryngeal nerve branching point; thus, they were identified as fascicles pertaining to the recurrent laryngeal nerve, separately from those coming from other visceral organs (Figure 2) (44). The precise distribution of fibers from the other visceral organs, especially fibers from peripheral cardiovascular targets, remains a matter of study, along with the definition of VN anatomical-functional models to guide the design of future VNS devices and protocols (48).

The VN is a mixed nerve with fibers carrying sensory, motor, and visceral information. It entails mostly afferent nerve fibers (80–90%) with fewer efferent fibers (10–20%) in the majority of mammalian species (26, 49). VN fibers are classified as “A-fibers,” “B-fibers” and “C-fibers” in accordance with the classical Erlanger/Gasser classification (50). Among the afferents, C-fibers are thin unmyelinated, Aδ-fibers are thin myelinated and Aβ-fibers are thicker myelinated. Among the efferents, Aα-fibers are the thickest myelinated axons of α-motoneurons that innervate pharyngeal and laryngeal muscles, while B-fibers are tiny, myelinated and carry parasympathetic inputs to visceral organs (51, 52). The diameters of the unmyelinated and myelinated fibers of the VN are in the range of 0.25–1.0 μm and 1–4 μm, respectively, in most animal species (53). Nearly all the large (above 10 μm) and 40–50% of the small (below 4μm) myelinated fibers are efferent (49).

Preganglionic parasympathetic fibers originate from the dorsal motor nucleus of the vagus in the brain stem, branch out of the VN main trunk to join several autonomic plexuses and synapse at cell bodies of postganglionic neurons, generally located in the wall of the target organ. Afferent fibers consist of the T-shaped axons of pseudounipolar sensory neurons, with their neuronal soma residing in the nodose and jugular ganglia (52–54). In the brainstem, central processes of jugular ganglion neurons project to the trigeminal nucleus through the spinal trigeminal tract (26), while the primary relay of vagal visceral inputs from nodose ganglion neurons is the nucleus tractus solitarii in the medulla (55, 56). The nucleus tractus solitarii has direct and indirect connections with a wide range of neural structures, thus endowing the VN with the control of a broad array of processes (Figure 1C) (55, 57, 58). An autonomic vagovagal loop encompasses visceral inputs to secondary neurons in the nucleus tractus solitarii, which then contact efferent neurons of the dorsal motor nucleus and sympathetic neurons of the rostral ventrolateral medulla to adapt the autonomic balance to physiological demands (Figure 1C) (3).

### Vagal Baroreceptors and Chemoreceptors

**FIGURE 3:**
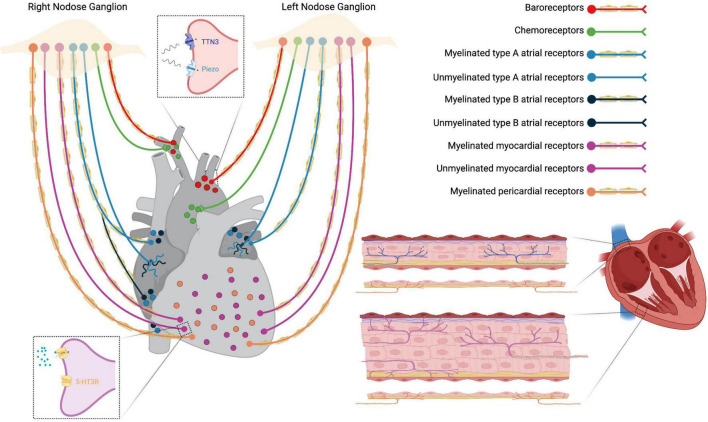
Schematic representation of thoracic cardiovascular afferent fibers running in the vagus nerve. Insets: top, baroreceptors Piezo2 + and TTN3 + terminals; down, unmyelinated 5-HT3R + myocardial receptors.

Vagal sensory neurons densely innervate great thoracic vessels and they include Piezo2 + /TTN3 + mechanosensory fibers, functioning as baroreceptors, and chemosensory fibers that detect arterial blood gas changes in the aortic bodies (Figure 3) (59). Afferent fibers from the left nodose ganglion innervate the apex of the aortic arch, while afferents from the right nodose ganglion innervate the right subclavian artery, near its branching from the innominate artery. These fibers run within the aortic depressor nerves forming fascicles that include both high-threshold mechanosensory and chemosensory afferents (59). The majority of vagal baroreceptors are myelinated fibers that convey information on stretch magnitude, pulse frequency and mean arterial pressure (59, 60) and, together with the carotid sinus innervated by the glossopharyngeal nerve, they represent the afferent arm of the arterial baroreflex (61–63).

### Vagal Cardiac Receptors

The VN provides both sensory and parasympathetic innervation to the heart *via* cardiac branches. Nodose neurons terminate with chemoreceptors and/or low-threshold mechanoreceptors in cardiac atria, ventricles, and major veins (Figure 3). Under normal circumstances, cardiac receptors are necessary for fine-tuning of the cardiovascular system. In CVDs like heart failure (HF), sensory endings undergo pathological activation that causes autonomic imbalance, with sympathetic excitation prevailing over vagal excitation (21, 22, 64).

There are at least two different types of atrial receptors belonging to the VN system of several mammalian species: type B receptors, that fire in response to increased volume (stretch receptors), and type A, that respond to atrial contraction. Both receptor endings correspond to slightly myelinated/unmyelinated fibers located mainly in the endocardium at the pulmonary veins-atrium and caval-atrium junctions and, to a lesser extent, in the free wall and appendage of both atria (65). They function as slowly adapting stretch receptors with low-frequency firing (66). Other vagal afferent fibers from the atria are unmyelinated C-fibers with a diffuse distribution and activity patterns similar to those described for type A or type B receptors (64).

Two types of sensory vagal endings have been described in both cardiac ventricles: myocardial and epicardial receptors (Figure 3). Myelinated myocardial receptors are mechanosensitive fibers working as tension/pressure-sensitive receptors and fire at the onset of left ventricular contraction (59, 64). On the other hand, unmyelinated myocardial receptors include 5-HT3R + C-fibers, predominantly functioning as mechanoreceptors (Figure 3) (64, 67), and C-fibers predominantly functioning as chemoreceptors which transduce the pain sensation that characterizes angina pectoris (21). Finally, vagal afferent fibers innervating the parietal pericardium are finely myelinated and sensitive to pericardium distension (64).

### Vagal Cardiac Efferent Fibers

The VN provides parasympathetic innervation to the heart *via* preganglionic cardioinhibitory neurons mainly located in the nucleus accumbens and to a lesser extent in the caudal dorsal motor nucleus of the vagus (21). Neurons of the nucleus accumbens possess thin myelinated axons with a diameter comprised in the B-fibers range (conduction velocity range 3–15 m/s) (68, 69) and exert strong respiratory- and cardio-modulatory and chronotropic effects. Efferent fibers from the nucleus accumbens of the right VN synapse with postganglionic cholinergic neurons that innervate the sino-atrial node, while fibers of the left VN project to postganglionic cholinergic neurons that innervate the atrioventricular node (21, 63). In contrast, neurons of the dorsal motor nucleus have unmyelinated axons, show little or no respiratory and cardiac modulation, exert smaller effects on HR and possibly stronger dromotropic and inotropic effects, as they project to postganglionic neurons innervating the left ventricle (70, 71). Cardiac neural control is realized *via* tonic interaction between sympathetic and parasympathetic limbs of the ANS, particularly in the mammalian heart, where many terminal fibers lie close to each other and exert reciprocal inhibitory effects at the synaptic level (21, 22, 72).

## Vagus Nerve Stimulation for Cardiovascular Diseases

Vagus nerve stimulation was first developed for the treatment of drug-resistant epilepsy and depression, obtaining FDA approval in 1994 (73–77) and 2005 (24, 78–81), respectively. Based on prior animal experiments, the first clinical studies defined the therapeutic range of VNS, the algorithm for stimulation titration up to the threshold of patient tolerance, and the safety and tolerability profile (73, 82). With the discovery of the inflammatory reflex in the early 2000s (83) and the evidence that VNS could attenuate inflammation normalizing the expression of proinflammatory cytokines (such as TNF-α and IL-6), VNS studies increased exponentially (28). At present, research labs worldwide are studying the effects of VNS in a multitude of conditions, spanning from neurological to inflammatory disorders, both in animal models and in patients (84).

**FIGURE 4:**
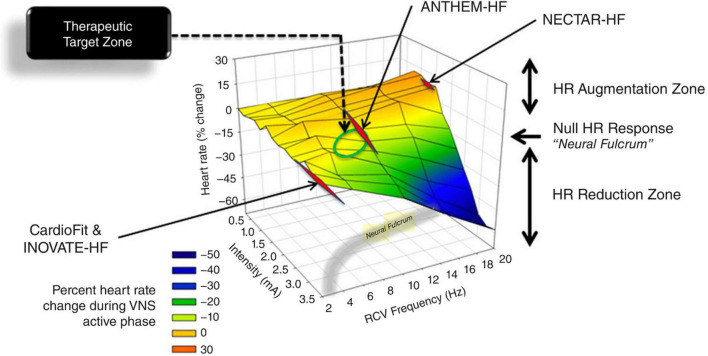
3D plot of percent change in heart rate against right VNS intensity and frequency, with representation of the neural fulcrum in the yellow-shaded region. Operational points used in the NECTAR-HF, INOVATE-HF, and the ANTHEM-HF studies. Adapted from Ardell et al. (17).

The VN can be stimulated in different ways and at different levels. The classical VNS consists in an invasive procedure that is performed as a day case procedure under general anesthesia. The FDA-approved VNS device comprises a spiral anchor and two bipolar helical electrodes with a platinum ribbon functioning as an anode and a cathode. They wrap approximately 270° around the left cervical VN, below the origin of the superior and inferior cervical cardiac branches, and are connected *via* a cable tunneled subcutaneously to a pulse generator that is most commonly positioned in a infra-clavicular pocket (24, 85–90). Despite the fact that VNS is a minimally invasive treatment, surgery remains inherently risky and comes with a number of possible side effects (91, 92). Therefore, alternative non-surgical methods have been developed, such as cervical non-invasive or transcutaneous VNS directed to the auricular branch of the VN (Figure 4) (91, 93–95). In the current standard practice, VNS parameters are set individually and tuned periodically for each patient. An “adequate” stimulation is generally set between a minimum level of perception by the patient to a maximum level of intolerability due to side effects, both of which are subjective and variable (96).

### Vagus Nerve Stimulation Mechanisms on the Cardiovascular System and Cardiovascular Diseases

Vagus nerve stimulation impacts cardiovascular control at multiple levels (97) *via* activation of afferent and efferent pathways and, depending on the frequency, pulse-width, and current intensity, of diverse fibers populations (43, 98).

In general, VNS of efferent cardiac fibers causes a reduction in heart rate (negative chronotropic effect on the sinoatrial node), in atrioventricular conduction (negative dromotropic effect on the atrioventricular node), and in ventricular contractility (negative inotropic effect on ventricular myocardium) (28, 99), with right VNS having mostly chronotropic effects while left VNS mostly dromotropic effects (100). VNS modulates left ventricular function increasing both action potential duration and the effective refractory period, either of which decreases intracellular calcium and ventricular contractility and wall motion (28, 89, 101). Activation of descending efferent projections can also mitigate sympathoexcitation *via* neural interactions within the intrinsic cardiac nervous system, modulate cardio-cardiac reflexes, and impart cardioprotection *via* direct effects on cardiomyocytes (1).

Vagus nerve stimulation of afferent fibers can impact central reflexes, including those that involve sympathetic and parasympathetic efferent outflows to the heart (1). For instance, VNS of vagal baroreceptors reflexively activates vagal cardioinhibitory efferent fibers to reduce heart rate and concurrently inhibits sympathetic efferent activity and down-regulates the renin-angiotensin-aldosterone system (28, 102).

Vagus nerve stimulation effects demonstrated beneficial effects in different animal models of CVDs. First of all, VNS has shown antiarrhythmic effects in several conditions, probably *via* multifactorial mechanisms that include a decrease in heart rate, the release of nitric oxide, anti-inflammatory effects, and antagonism of the sympathetic nervous system (28, 103). It was shown that VNS increases the threshold for ventricular arrhythmias *via* reduction in ventricular excitability and repolarization heterogeneity (effect on ventricular conduction system) (28, 99). In animal models of atrial fibrillation, VNS exhibited antifibrillatory effects by shortening atrial fibrillation duration and prolonging the atrial fibrillation cycle length (19, 28). Moreover, a VNS delivered below the threshold of bradycardia induction can effectively suppress atrial fibrillation in anesthetized dogs (89). VNS effects on the sympathetic nervous system contribute to the prevention of arrhythmias also during cardiopulmonary resuscitation (28). VNS-induced decrease in cardiac motion reduces cardiac metabolic demands during the vulnerable period of ventricular fibrillation, making VNS a potential intervention to improve the efficacy of defibrillation (101).

Vagus nerve stimulation was also shown to decrease infarct size and to halt post-myocardial infarction phenomena such as the remodeling of both the myocytes and the intrinsic cardiac neuronal system. These cardioprotective mechanisms include anti-inflammatory effects, prevention of Connexin 40 and Connexin 43 loss, antioxidative effects, and antiapoptotic effects such as decrease in cytochrome *c* release and in the proapoptotic Bcl-2-associated X protein levels (28, 89, 103–106). When applied during myocardial reperfusion, VNS was shown to improve ventricular function and reduce arrhythmic episodes *via* antagonization of the cardiac sympathetic outflow, reduction of reactive oxygen species and of ventricular excitability (28, 103–106). VNS improves left ventricular ejection fraction post-myocardial infarction restoring subcellular levels of calcium-binding proteins (such as SERCA2a, NCX1, and PLB) and can reestablish baroreceptor reflex to the pre-infarction baseline (28).

Vagus nerve stimulation can slow the progression of myocardial remodeling and atrial and ventricular dysfunction in animal models of chronic HF with reduced ejection fraction (28). VNS beneficial effects in HF can be attributed to improvements in left ventricular mechanics, attenuation of the sympathetic drive, down-regulation of the renin-angiotensin-aldosterone system, reduction of proinflammatory cytokines, normalization of the nitric oxide pathway, increase in myocardial expression of gap junction proteins and capillary density and tempering of myocardial interstitial fibrosis (1, 28, 104, 106–113). Optogenetic stimulation of cardioinhibitory neurons in the dorsal motor nucleus of the vagus can reduce myocardial expression of G-protein-coupled receptor kinase 2 (GRK2) and b-arrestin 2, which both contribute to the progressive decline of myocardial contractile function in HF (104).

Vagus nerve stimulation can ameliorate poststroke recovery *via* enhancement of motor cortex plasticity during rehabilitation, likely favoring the release of acetylcholine, norepinephrine, GABA, and brain-derived neurotrophic factor (28). VNS can also attenuate cerebral edema after brain injury by reducing cerebral blood flow, glutamate excitotoxicity, and inflammation (28).

Finally, VNS in hypertensive rats showed a significant blood pressure reduction, with static stimulation clinically more effective than pulsatile stimulation (28).

### Vagus Nerve Stimulation in the Clinical Scenario

To date, despite the vast assortment of CVDs investigated in the pre-clinical scenario, VNS clinical applications in the cardiovascular field have been mostly focused on HF. This syndrome provides a strong rationale for ANS modulation, as its genesis and progression are heavily influenced by autonomic imbalance (21, 22, 114, 115). Preclinical studies have shown that VNS can exert very positive effects on the progression of HF, but clinical trials failed to achieve the same results. Complete clinical trials of VNS for HF with reduced ejection fraction include two randomized controlled trials, i.e., the INOVATE-HF (116) and the NECTAR-HF (117), and two open-label studies, i.e., the ANTHEM-HF (118) and the study by De Ferrari et al. (119). In the study by De Ferrari et al. (119) and in the INOVATE-HF, VNS was delivered using the CardioFit system that senses heart rate (*via* an intracardiac electrode) and delivers asymmetric stimulation at a variable delay (70–325 ms) from the R-wave (85, 87, 119, 120). The stimulation lead is an asymmetric bipolar multi-contact cuff electrode specifically designed for cathodic induction of action potentials while simultaneously applying asymmetrical anodal blocks, thereby reducing the activation of A-fibers while preferentially activating efferent B-fibers (85, 87, 105). In the NECTAR-HF (117) and the ANTHEM-HF (118) trials the investigators utilized the Boston Scientific VNS device that activates VN fibers bidirectionally with no synchronization with the cardiac cycle (118). All trials recruited similar NYHA class II-III patients with reduced left ventricular ejection fraction and receiving optimal medical therapy (121). These trials did not raise safety issues but showed variable efficacy (116, 118, 121, 122). As highlighted by a recent meta-analysis, these trials showed significant improvement in the functional NYHA class, quality of life, 6-min walking test, and NT-proBNP levels, but VNS did not have any impact on mortality (123). The subjective measures that improved in all trials should be cautiously taken as subjects were not totally blind to the therapeutic procedures, notwithstanding the sham-controlled design (116, 118, 124). Only the two uncontrolled studies (ANTHEM-HF and the study by De Ferrari et al.) showed a positive effect of VNS on cardiac remodeling (118, 119), despite the success of preclinical experiments (118, 120). These trials employed diverse stimulation parameters, and subsequent analyses showed that the ANTHEM-HF study was the only one to achieve the therapeutic stimulation corresponding to the “neural fulcrum” (17). The neural fulcrum is defined as the combination of VNS parameters such as frequency–amplitude–pulse width that results in no heart rate response (Figure 4), and it corresponds to a dynamic equilibrium where VNS activates cardiac neural circuits while keeping reflex control of cardiovascular functions. In fact, VNS normally modifies cardiac neural circuits pushing them in one direction that tends to be physiologically counterbalanced by cardiovascular reflexes (17). For instance, low intensity/high frequency (20 Hz or more) stimulation preferentially activates afferent fibers causing tachycardia that leads to secondary activation of both central pathways and vagovagal, vagosympathetic, or vagoadrenal reflexes. On the other hand, higher intensity/lower frequency (10–15 Hz) stimulation activates parasympathetic neurons resulting in bradycardia and mitigation of sympatho-excitation *via* neural interactions within the intrinsic cardiac nervous system (1). Finally, low intensity/very low frequencies (1–2 Hz) stimulation determines little to no cardiomotor effects, as the afferent-driven decreases in central parasympathetic outflow are equivalently counteracted by direct activation of cardiac parasympathetic neurons (125).

ANTHEM-HF utilized the principle of neural fulcrum by determining the autonomic engagement *via* an automatic beat-to-beat pattern analysis throughout the initial phase of VNS titration (122). In the NECTAR-HF trial, the high frequency stimulation provoked patients intolerance and impeded titration to a therapeutic dose; in the INOVATE-HF study, not all patients received adequate stimulation levels (116, 121, 122, 126–128). On the basis of the results of ANTHEM-HF, a large, randomized, controlled trial of right VNS with the use of the same system is now underway (ANTHEM-HFrEF PIVOTAL trial, NCT03425422) (18), along with a novel study in patients suffering from HF with preserved and mid-range ejection fraction (127).

### Unresolved Issues in Clinical Vagus Nerve Stimulation for Cardiovascular Diseases

The exploitation of VNS full potential and its transformation into a simple and cost-effective therapy for a wide range of conditions requires the completion of some major steps. A general problem is the still limited knowledge of VN function, with an ensuing lack of understanding of the mechanisms responsible for already established VNS treatments of diseases such as drug-resistant epilepsy (121, 126, 129). Consequently, optimum stimulation parameters tailored for patient-specific clinical characteristics and precise timing remain a matter of debate (74, 75, 79, 128, 129). VNS is normally up-titrated through a series of follow-up visits until a therapeutic dosage is attained without adverse effects and up to the tolerance threshold of the patients (73, 94). Given subjectivity of patients’ tolerance and the uniqueness of the electrode-nerve interface, no standard therapeutic dose exist, the effectiveness of parameters adjustment during titration remains dubious, and the response prediction uncertain (92, 121, 122, 129, 130).

Another related problem is the definition of the target population using adequate “predictors” to discriminate between responders and non-responders. These predictors could be markers of autonomic imbalance represented by physiological parameters such as heart rate variability or innovative markers such as those derived from neural decoding of specific ANS circuits (78, 118, 129, 131).

While technology advances at a quick pace, neuromodulation’s ultimate potential can be realized when the relationship between nerve activity and physiological function is thoroughly known (4, 6), thus allowing translation of biological information into appropriate engineering specifications (16, 132). The knowledge gap of vagal physiology, functional anatomy and neuromodulation mechanisms inevitably also affects the selectivity of neural interfaces and of neuromodulation protocols (133). First of all, most VNS systems lack functional selectivity, that is stimulation of distinct functional classes of fibers, and are far from mimicking patterns of action potential occurring in healthy nerve fibers (32, 133). Secondly, most VNS systems lack spatial selectivity, that is selective modulation of fibers in the specific anatomical territory innervated by a given fascicle (32). Electrodes commonly used for VNS are not selective enough to achieve targeted neuromodulation in a complex fasciculate nerve like the VN (133). The direct consequences are the failure to achieve therapeutical effects and the onset of side effects that include hoarseness, throat pain, voice alteration, difficulty swallowing, coughing, abdominal and chest pain, nausea, dyspnea, and bradycardia (36, 85–88, 90). The inadvertent stimulation of somatic nerve branches such as the superior and recurrent laryngeal nerve has been addressed as one of the main causes of VNS side effects (36). Selective stimulation of vagal cardiac B and C fibers can be challenging given that their thresholds are 2–100 times greater than A fibers, as those branching to the laryngeal nerves (89). To achieve such selectiveness, several authors tried the combination of different stimulation parameters or to modify the pulse shape using different techniques such as the anodal block, slowly rising pulses or depolarizing pre-pulses (89). Other strategies are the modification of electrode design to allow preferential activation of efferent fibers (such as in the case of the CardioFit system) or the development of multicontact electrodes that exploit the topographical architecture of human nerves to target organ-specific fascicles (36, 88–90). This last approach would provide better spatial resolution and consequently improve the selectivity both for recordings and stimulation (36, 89). Therefore, new neural interfaces with higher electrode counts and spatial selectivity should be implemented (8, 30), along with advanced signal processing techniques and the use of hybrid models (134, 135) with extensive validation in experimental animals (136). With such technologies, the development of closed-loop VNS based on the combination of selective nerve stimulation and biosensing technologies could be one of the best solutions to overcome the aforementioned limitations emerged during VNS clinical trials (7, 133).

### Closed-Loop Strategies for Vagus Nerve Stimulation

The current VNS systems provide stimulation in an open-loop fashion, meaning that parameters are pre-set and are not automatically adjusted according to the patient’s clinical characteristics (137). Closed-loop stimulation strategies offer the advantage of providing treatment only in response to detection of altered biomarkers of disease, thus tuning the stimulation according to the patient’s condition (138, 139). This approach potentially improves the efficacy of open-loop interventions and decreases the associated side effects (140). Closed-loop devices should continuously monitor internal biological variables to adjust therapy to individual conditions (7), thus allowing not only automatic but also adaptive neurostimulation that could maintain its efficacy over time and overcome the intrinsic plasticity of biological systems (8, 11, 141). In fact, plasticity and memory are crucial characteristics of the cardiac neuraxis, which undergoes profound alterations in chronic CVDs, causing the disruption of homeostatic cardiovascular functional responses (19).

**FIGURE 5:**
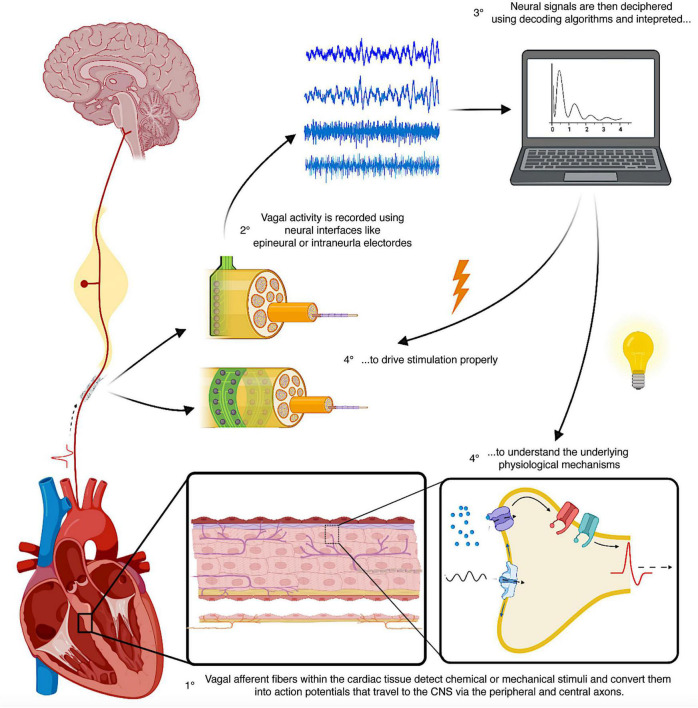
Schematic representation of the principles of closed-loop neuromodulation using neural recordings and decoding strategies. The heart is densely innervated by vagal afferent fibers, which detect chemical and mechanical stimuli, convert them into electrical signals (action potential) and send them to the CNS *via* their peripheral and central axons. Using neural interfaces placed on the vagus nerve, it is possible to record and decode these electrical signals and identify the underlying physiological processes and set the spatiotemporal characteristics of stimulation. CNS, central nervous system.

Closed-loop VNS relies heavily on the precise selection and processing of physiological inputs (32, 142). To date, in the clinical scenario, only macro-biosignals like heart rate have been employed as input data for control loops. This concept is well illustrated in the work by Tosato et al. who achieved heart rate regulation with a closed-loop control system that continuously measured the RR interval, recalculated the difference between the measured and the target value and fed it back to the stimulator accordingly (100). Multiple other indirect and non-invasive measures can be used as indexes of cardiac VN activity (115), for instance heart rate variability, baroreflex sensitivity or respiratory sinus arrhythmia (21, 115). However, such clinical vagal indexes should be used with caution as they represent gross markers of the final net effect of parasympathetic and sympathetic action on the heart (72). On the other hand, local control loops can be obtained by recording feedback bio-signals from the same spot where the stimulus is delivered (142). This approach optimizes the spatial and temporal distribution of the local stimuli (142) but requires high-fidelity feedback signals that are not clinically obtainable due to current technological limitations (142, 143). A good example is represented by the lack of instruments to properly follow and locally evaluate in real-time the myriad of metabolic signals or the fluctuating levels of inflammatory stimuli. Nevertheless, all this information is collected by the peripheral sensors of the ANS and converted into electrical signals (neural encoding), a sort of “neural footprints” of physiological processes that can be recorded and decoded (Figure 5) (8, 30). Deciphering the neural language through decoding techniques would be essential to understand the underlying mechanisms of many diseases and to develop new methods and technologies that better engage with neural circuities (141). In the case of VNS, the copresence of afferent and efferent fibers in the VN offers the opportunity to build a feedback loop on the same anatomical site, that is to record from and then to stimulate the VN (Figure 5) (144, 145). Vagal sensory neurons are equipped with a vast arsenal of receptors to sense and respond to a huge variety of stimuli (27). Consequently, neural signals traveling through the VN represent a peerless source of information, and this helps the implementation of neural decoding strategies that usually benefit from an adequate number of signals (30, 32). Moreover, vagal neural signals offer the advantage of high temporal and spatial resolution (146), helping in the identification and classification of patterns difficult to see using other biosignals (8, 30) and potentially allowing devices to diagnose various conditions before symptoms presentation (32). New neural interfaces with higher electrode densities and spatial selectivity, such as intraneural electrodes, (147) and advanced signal processing techniques (8) will be necessary to take full advantage of the information traveling along with the VN. In the next section, we present the main preclinical studies that aim at the development of VNS closed-loop approaches based on different neural recording and decoding strategies for CVDs.

#### Decoding Techniques for Physiological Fiber Firing

Neural interfaces used for stimulation can also be used to record neural signals and monitor ANS activity in real-time (13). While innovative neural interfaces with multiple contacts are designed to improve the quality and information content of neural recordings (148–151), comparable efforts are being made to develop advanced signal processing and data analysis methods (152, 153). Several studies recently focused on the extrapolation of neural markers from spontaneous or physiologically enhanced VN activity, employing various decoding techniques. One technique is the coherent electroneurogram averaging that aims at the isolation of the neural activity of interest from the random noise by taking the average of N snippets from a recorded signal in correspondence to an external or an internal trigger, for instance a biological change (154). Using multicontact cuff electrodes, Plachta et al. employed the ECG rising edge as a trigger to remove stochastic noise, isolate baroreceptors activity from VN recordings and perform selective VNS reducing blood pressure without producing side effects in rats (145). Sevcencu et al. recorded signals from the porcine left VN to extract intraneural and extraneural profiles resembling the temporal evolution of blood pressure during baseline activity. In particular, systolic peaks and dicrotic waves characterizing blood pressure were reflected by the neural counterpart (144, 155). Rozman and Ribarič employed 33-electrode spiral cuff to record from the left VN of a dog during stimulation of cardiovascular or respiratory stimulations and identified the channel that was best correlated with heart activity using the spectrum estimation technique (156).

Another common approach to extract physiological information from VN electrical activity consists of decoding fiber spike patterns using spikes sorting techniques (157–162). Typically, raw neural signals are band-passed from 200 Hz to a maximum of 10 kHz, robustly denoised, spikes are then detected using thresholding methods and clustered using feature waveforms (46, 163). In general, there is not a universally adopted low-frequency cutoff as it usually depends on the quality and the nature of the signals. For instance, 200 Hz low-frequency cutoff was used in the context of intrafascicular sciatic nerve recordings (164), 1 kHz for intraneural recordings from the pig VN (136), 700 Hz (165) and 300 Hz (166) in the case of microneurographic recordings from the peroneal nerve and from the human VN, respectively. In the murine VN, spike sorting techniques within decoding frameworks have been employed to decode the activity of different fiber types enhanced by inflammatory stimuli such as particular cytokines (153) or metabolic ones like hypoglycemia/hyperglycemia (167). Spike sorting techniques are potentially usable in human patients as we recently obtained single-fiber recordings from the human cervical VN identifying tonically active neurons that discharged synchronously with the respiratory and cardiac cycles (166). Spike-like signals, as they reflect the activity of individual fibers, are preferable to cumulative signals to obtain maximal functional selectivity (162). Real-time implementations of complex spike sorting algorithms onto low-power off-the-shelf digital signal processors are currently available, as in the case of neuroprosthetic applications where the power consumption enabled more than 24 h processing at the maximum load (162). In the case of closed-loop VNS protocols, this would allow longer operational time scales, such as Holter-like monitoring at a neural level, i.e., a “Neural Holter” with biomarkers extracted directly from neural activity. As pointed out in Raspopovic et al. (136), signals recorded with intraneural electrodes can be classified as a hybrid category between cumulative and single-unit signals. This characteristic allows the development of more robust recording schemes and processing algorithms *via* a combination of decoding strategies developed on both cumulative and single-unit signals (136).

**FIGURE 6:**
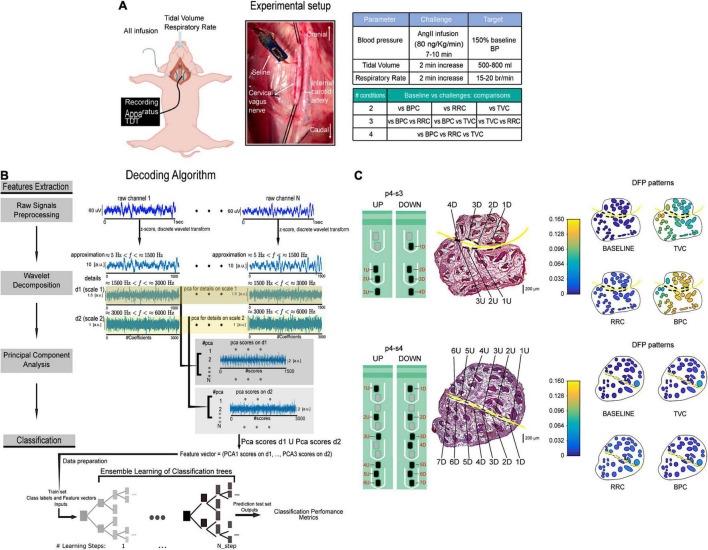
Schematic representation of the experimental setup and decoding algorithm from Vallone et al. (152). **(A)** Recording apparatus, electrode implantation and summary of the *in vivo* protocol and functional challenges comparisons. **(B)** Decoding algorithm. Feature extraction was performed on 1 s portions of raw intraneural signals (dark blue traces) by applying principal component analysis on wavelet details relative to two different scales (1,500 Hz < *f* < 3,000 Hz and 3,000 Hz < *f* < 6,000 Hz). Neural signals recorded with intraneural electrodes were a combination of single-unit and compound action potentials. Decoding performed with ensemble learning based on classification trees combined with random undersampling and boosting procedure was applied on feature vectors. Decoding performance was assessed by means of confusion matrices and accuracy level. **(C)** Schematic description of two intraneural electrodes implantation in the VN of one pig and hybrid model simulations of fascicles activation at baseline and in response to functional challenges. The two intraneural electrodes are represented on a VN histological section (central panels). DFP patterns of activity during baseline and each functional challenge (left panels). DFP, discriminative field potentials. Adapted from Vallone et al. (152) with permission.

The identification of neural signals elicited by specific physiological stimuli could be extremely useful to distinguish among VN fibers coming from different cardiovascular sites and carrying information on multiple functional parameters that can vary over short time windows, such as cardiac output and blood pressure. Such distinction could be further appreciated using neural multielectrode devices with high spatial selectivity to better interface the potential topographical architecture of the VN (44, 46). However, cuff electrodes can only sense compound nerve action potentials and multi-unit activity and they do not allow access to single-fiber action potentials contrary to intraneural electrodes (89, 168). Intraneural electrodes offer the advantage of higher signal-to-noise ratio and higher spatial specificity compared to epineural electrodes and could allow more effective closed-loop decoding methods to be used (8, 139, 169). Intraneural electrodes such as the Longitudinal Intra-Fascicular Electrode (LIFE) or the Transversal Intra-Fascicular Multi-channel Electrodes (TIME) have provided rich and valuable sensory feedback in human amputees and detailed information from decoding hand movements in somatic nerves (139, 157, 159, 170). The LIFE is a flexible electrode consisting of 25–50 μm diameter Pt or Pt-Ir wires insulated with Teflon or metalized Kevlar fibers insulated with medical-grade silicone. The wire is surgically inserted into the nerve along the fascicle and then pinched out of the nerve again. The recording sites are areas of 0.5–1.5 mm long which are left uninsulated (149). A more recent version of LIFEs is the thin-filmLIFEs (tfLIFE), based on a thin highly flexible micropatterned polyimide substrate filament that can host eight contact sites (46, 149). The TIME consists of a thin, strip-like polyimide substrate with platinum electrode sites. The substrate is folded to align several electrodes and the folded substrate is threaded transversely through the nerve between the fascicles (149). The original design contained 10 sites with interelectrode spacing of 230 μm (148). The TIME was developed to achieve good contact with nerve fibers, selectively addresses several fascicles in a nerve with a single implant, and minimizes the mechanical mismatch between the implanted material and nerve tissue (168). The TIME has shown higher selectivity at low stimulation intensities than the single LIFE and multipolar cuffs (46, 148, 149). Our group combined the use of multichannel intrafascicular electrodes, machine learning principles and hybrid models (136) to study high frequency (>1,500 Hz) VN activity of anesthetized pigs during artificially produced alterations of physiological parameters, simulating increases in respiratory rate, tidal volume and arterial blood pressure (Figure 6A) (152). Using a new decoding algorithm that combines wavelet decomposition, dimensionality reduction, and ensemble learning classifiers, we could associate VN signals to specific functional changes (Figure 6B). Our approach was a machine learning-driven approach to find informative feature vectors for reliable decoding of cardio-respiratory alterations regardless of their precise nature, and future analysis will serve to get more interpretable features related to units and aggregate activities. Different from epineural electrodes that can only provide a global picture of neural signal trafficking (147, 171), we employed intraneural electrodes to enhance selectivity (147, 169) and to map a possible spatial functional organization of VN fascicles. Thus, we employed a hybrid modeling framework based on histological analysis combined with electrode discrimination ability properties measured *via* a novel quantitative measure called Discriminative Field Potential (DFP) and we obtained distinct spatial configurations of discriminative patterns generated by fascicles during the various functional challenges (Figure 6C) (152). This is extremely important for the development not only of timely, but also spatially selective stimulation protocols in a complex nerve with multiple fascicles like the human or porcine VN (152). In this perspective, the precise knowledge of cardiovascular fibers arrangement within the cervical vagal trunk would be crucial to establish effective VNS protocols for the treatment of CVDs. Anatomical models and non-invasive tests should be developed and used to better anticipate which site for neuromodulation would give the best outcomes and to overcome the anatomical variability that limits clinical VNS applications (116, 120, 129).

The biocompatibility and longevity of intraneural electrodes was demonstrated in animal models and preliminarily confirmed in human experiments with long-term stimulation for sensory feedback and chronic neural recordings (139, 172). However, the experience in chronic implantations in humans indicates that there is frequently a reduction in the number of functioning electrode active sites, an in- crease in the stimulation threshold, and a decrease of the signal-to-noise ratio along time. To further improve the usability of the neural electrodes, considerable efforts are being devoted in the engineering field for increasing robustness and flexibility at the same time of miniaturizing the electrodes and in the biological field to increase biocompatibility of the substrates and to modulate the foreign body reaction (46). On the computational side, since the drift in the amplitudes of signals and changes in the signal-to-noise ratio greatly hampers chronic neural recordings and decoding, new algorithms for drift compensation have been developed (173). In the case of the VN, carbon nanotube yarn electrodes have been used to make the first direct chronic measurements of vagal tone in freely moving rats (174). Thanks to their small size, high flexibility, and low impedance, carbon nanotube yarn electrodes have provided stable, high-signal-to-noise chronic recordings in rats VN with high-quality signals continuing up to 4 months after implantation (174).

#### Decoding Fiber Activity in Electrically Stimulated Nerves

Recording and decoding the spontaneous activity of vagal fibers would be useful to determine the precise timing for VNS delivery, but may prove limited for the definition of precise dosing (133). In this regard, recording and decoding fiber activity during VNS may represent a complementary method to better define the relationship between stimulation and physiological effects.

The standard VNS dosing method does not rely on any measurement of fiber activation, since the commercial implants lack the capability to record from nerves during stimulation. Consequently, factors like electrode interface and nerve sensitivity are not controlled and VNS effects in a patient are neither uniquely determined over time nor comparable with other patients (133). The ability to distinguish between stimulated fibers could aid in correlating neural activity to external variables, thus increasing the ability to achieve targeted stimulation (175), with decreased variability in therapeutic responses and increased response rate (96).

**FIGURE 7:**
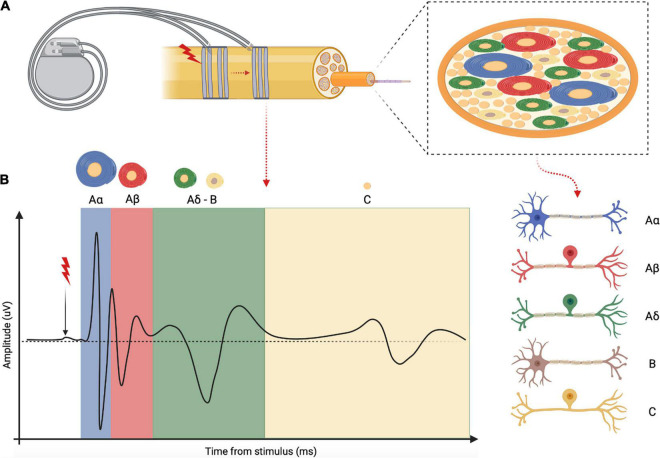
Schematic representation and explanation of the electrically evoked compound action potential (eCAP). **(A)** Example of a VNS device that electrically stimulate the vagus nerve at one site while recording evoked activity at another site. Inset: vagal fascicle containing different fiber types distinguished by myelination degree and fiber caliber. **(B)** Example of a complete vagus nerve eCAP obtained with supra-threshold stimulation of all vagal fibers subtypes. Each peak of the eCAP corresponds to different fiber subtypes as indicated by the different color codes.

In studies that employ extraneural interfaces, nerve activity is frequently analyzed in terms of evoked compound activity by electrical stimulation (eCAP) (176). The diverse conduction velocities of various fiber types, which ultimately depends on fiber caliber and degree of myelination, determine typical patterns and shapes with distinctive latencies and peaks in the eCAP (177), as shown in Figure 7. A sophisticated analysis of VN eCAPs would help in the assessment of the relationship between stimulus dose, neural recruitment and physiological effects (178).

Tahry et al. obtained, for the first time, VN eCAPs recordings after implanting the Advanced Nerve Stimulator version 300 (ADNS-300, Neurotech SA, Louvain-La-Neuve, Belgium) in the human VN (179). In preclinical studies, VNS-eCAP were used to optimize stimulation parameters and electrode design during VNS in dogs (176), pigs (180), and rodents (96), showing a strong correlation with the physiological effects of stimulation. For instance, vagal B-type fiber eCAP amplitude was correlated with changes in heart activity (176), indicating that parasympathetic B-fibers are the best predictors of cardiac activity during VNS (96, 177). Ordelman et al. found an indirect component in pig vagal eCAPs during VNS protocols, and they showed that it correlated with the state of the cardiovascular system (181).

## Conclusion

**FIGURE 8:**
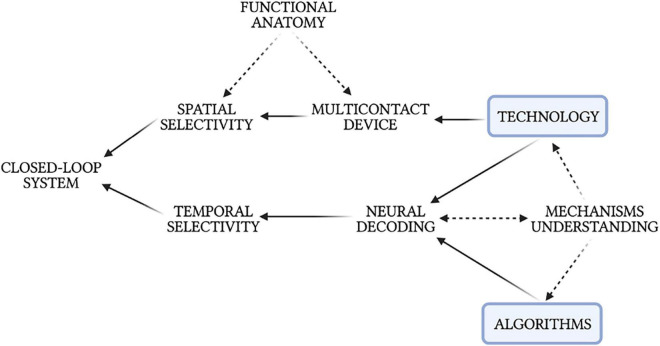
Intercorrelations between neural decoding, technology, algorithms, physiological mechanisms, and the development of a closed-loop neuromodulation system.

In conclusion, the VN represents a key component of the cardiac neuraxis and VNS has shown a great potential for the treatment of a wide range of CVDs in the preclinical setting. Results in experimental animals may not be immediately translated into clinical applications, yet they are paving the way for fine-tuning and customized VNS applications. VNS represents a cheaper alternative/complementary solution to pharmacological remedies that lack of efficacy and present significant side effects and astronomical costs (4, 7, 182, 183). Closed-loop VNS would guide earlier, more accurate diagnosis and enable more effective, less costly prevention and intervention compared to pharmacological treatments (183). Moreover, access to personalized bioelectronic data would facilitate greater patient understanding of their conditions and greater engagement with their treatments, higher levels of health literacy, and greater communication and trust between patients and physicians (183). A plethora of promising research is advancing to overcome VNS limitations and develop closed-loop modalities. Advanced neural decoding strategies represent a major candidate. However, automatic closed-loop modalities will require not only advancements in biotechnologies but also improvements in the basic understanding of fundamental biological mechanisms (16). Progress in neural interface technology, big-data analysis methods, and signal processing techniques will accelerate biological breakthroughs that, in turn, will inform additional advancements in technology and methodology, creating a synergistic loop that ensues cross-disciplinary collaboration (Figure 8) (8).

## Author Contributions

MMO conceptualized, wrote the manuscript, and prepared the figures. FV wrote and revised the manuscript. SM and FR conceptualized and revised the manuscript. All authors authorized the submission of the manuscript.

## Conflict of Interest

The authors declare that the research was conducted in the absence of any commercial or financial relationships that could be construed as a potential conflict of interest.

## Publisher’s Note

All claims expressed in this article are solely those of the authors and do not necessarily represent those of their affiliated organizations, or those of the publisher, the editors and the reviewers. Any product that may be evaluated in this article, or claim that may be made by its manufacturer, is not guaranteed or endorsed by the publisher.
